# Comparative Aspects of Osteosarcoma Pathogenesis in Humans and Dogs

**DOI:** 10.3390/vetsci2030210

**Published:** 2015-08-17

**Authors:** Timothy M. Fan, Chand Khanna

**Affiliations:** 1 Department of Veterinary Clinical Medicine, Comparative Oncology Research Laboratory, University of Illinois at Urbana-Champaign, Urbana, IL 61820, USA; 2 Tumor and Metastasis Biology Section, Pediatric Oncology Branch, Center for Clinical Research, The National Cancer Institute, Washington, DC 20004, USA; E-Mail: ckhanna@animalci.com

**Keywords:** bone sarcoma, comparative oncology, conserved pathogenesis, spontaneous tumor modeling

## Abstract

Osteosarcoma (OS) is a primary and aggressive bone sarcoma affecting the skeleton of two principal species, human beings and canines. The biologic behavior of OS is conserved between people and dogs, and evidence suggests that fundamental discoveries in OS biology can be facilitated through detailed and comparative studies. In particular, the relative genetic homogeneity associated with specific dog breeds can provide opportunities to facilitate the discovery of key genetic drivers involved in OS pathogenesis, which, to-date, remain elusive. In this review, known causative factors that predispose to the development OS in human beings and dogs are summarized in detail. Based upon the commonalities shared in OS pathogenesis, it is likely that foundational discoveries in one species will be translationally relevant to the other and emphasizes the unique opportunities that might be gained through comparative scientific approaches.

## 1. Introduction

Osteosarcoma (OS) is a malignant tumor derived from primitive mesenchymal stem cells with the capacity to produce osteoid matrix [1,2]. In pediatric patients, this is a disease of the second decade in life, with the highest incidence following peak adolescent growth. Consistent with this potential connection to bone growth, OS in dogs disproportionately affects large and giant breeds, and has been connected to dysregulation of the Insulin-like Growth Factor-1-Growth Hormone (IGF-I-GH) axis [3,4,5]. Consistent with its stem cell derivation, OS in both species can adopt a spectrum of cellular phenotypes of mesenchymal differentiation including, cartilage, bone, fat and muscle. Not surprisingly, histologic variants of OS include a variety of mesenchymal forms including chondroblastic, fibroblastic, osteoblastic, and telangiectatic subtypes [6]. Clinically in both dogs and children, OS develops predominantly in the metaphyseal regions of weight-bearing bones, most commonly the distal femur, proximal tibia, and proximal humerus [7]. A strikingly consistent feature of OS biology in both species includes the high risk for metastatic spread to the lung. Indeed, for both species, understanding the biology of metastasis and using this knowledge to develop novel therapeutic options is critically important [8]. The incidence of OS in human patients is bimodal, affecting both pediatric and geriatric age groups. The first and largest peak, as noted above, occurs during adolescence between the ages of 10–19 years, and smaller peak of OS occurs in adults older than 65 years, often associated with Paget’s disease [9]. The overall incidence of OS is five per million cases at risk per year in the adolescent population, resulting in approximately 800–1000 children in the United States developing OS annually [10]. In dogs, the prevalence of OS is notably higher, and estimated to be at least 10-fold greater than of humans, with approximately 10,000 dogs a year in the US developing OS based upon the number of resident canines and reported incidence rate [11].

Biologically, OS originates within the intramedullary cavity of metaphyseal bone. The growth and progression of appendicular OS result in the effacement and erosion of the immediate bone microenvironment, including the marrow cavity and circumferential cortical and trabecular bone. In addition to localized skeletal perturbations, OS metastasizes to distant visceral organs, most commonly the pulmonary parenchyma via hematogenous dissemination [12,13,14]. For OS patients, control of the primary tumor can be provided through a number of effective surgical approaches. However, despite complete control of the primary tumor, metastatic spread to the lungs continues to be a problem. The progression of metastases has been reduced with adjuvant chemotherapy, yet a substantial fraction of patients still succumb to distant metastases. Collectively, this combination approach has improved the long-term survival of pediatric patients from approximately 20% (surgery alone) to over 60% (surgery with adjuvant chemotherapy) [15,16]. Unfortunately, approximately 30%–40% of pediatric OS patients will subsequently develop recurrent disease in the form of distant metastases, and dose intensification of adjuvant systemic chemotherapy achieves little, if any, additional survival improvement for this subset of patients [16]. The prognosis for elderly patients diagnosed with OS remains poor regardless of therapeutic interventions, with the five-year survival rate of less than 20% for OS associated with Paget’s disease [17,18,19,20]. The magnitude of benefit associated with chemotherapy is similar in dogs; although it is reasonable to consider the disease to be more aggressive in dogs than people [21]. Given the more aggressive biology of canine OS, dogs treated with multimodality therapies still experience an exceeding high rate of mortality (~85%–90%) within two years of diagnosis as a consequence of disease recurrence in the form of distant metastases. 

A unifying pathogenesis for the development and progression of OS remains to be defined in either human or dog. The described similarities in the biology of the disease between both species suggest the potential opportunity to clarify OS pathogenesis through a comparative approach that studies the disease in both species. The value of such a comparative approach is reinforced though the consistent finding in both species of complex genetic landscapes (*i.e.*, markedly aneuploid karyotypes) in this cancer. Indeed, the extreme complexity of the OS cancer genome is such that it is unlikely that recurrent genetic alterations will be identified through the study of one species alone. Indeed, this is in fact the experience in genomic studies conducted to-date in both species. These unrewarding genetic studies conducted in isolation might only be improved through the cooperative use of one species as a genetic “filter” to aid the study of the other species. The implementation of such a discovery strategy is likely to increase the chances for identifying common variants that might otherwise be missed in the study of a single species [22,23]. The complexity of the OS landscape has also suggested the value in longitudinal studies that may uncover the earliest genomic risk factors for the development of OS. This longitudinal approach to study OS may answer what early genetic events are needed to permit the survival of cancer cells with such bizarre genetic aberrations. Indeed, conventional wisdom based upon molecular biology underpinnings would suggest that such alterations should not be tolerated by a cell, and accordingly should result in apoptosis of such genetically altered cells. A reasonable extension of this rationale may be to ask if an early alteration in DNA repair or surveillance functions may underlie the development of OS, and accordingly may prioritize the study of germline alteration as part of a longitudinal study of OS progression. As a start, the study of such germline risk factors for the development of OS has recently been examined. The etiopathogenesis of OS in humans is complex in the form of genetic studies in both the dog and the human [24,25,26,27,28,29]. Specifically derived from comprehensive gene wide association studies (GWAS) in people, two loci were identified to be associated with risk for OS development, including, *GRM4* gene at 6p21.3 (encoding glutamate receptor 4; *p* = 8.1 × 10^−9^) and a locus in a gene desert at 2p25.2 (*p* = 1.0 × 10^−8^) [30]. Biological studies into the mechanisms by which these loci may contribute to sarcomagenesis are needed. It is unclear if there is any connection between these loci and the observation of the complex genetic landscapes of OS or the mechanisms by which such genetic dysregulation is tolerated in a cell. The finding of an association of OS risk within a “gene desert” will clearly require further studies, and potential analysis of regulatory roles associated with this desert. In the dog, a recent GWAS identified 33 OS-associated loci [28]. Pathway analyses of human genomic regions with synteny to the canine loci revealed functional connections of these related to growth, osteoblast differentiation and proliferation, and tumor suppression. Only a fraction of the genes and pathways reported in the canine GWAS have been previously implicated in OS, further demonstrating the potential value of a comparative GWAS approach, especially in the setting of a rare disease like OS in humans. Some discrepancies were identified between human and canine studies, as GRM4 was not specifically identified as was the case in the human GWAS; however, another glutamate receptor gene, GRIK4, was significantly associated in subsets (defined by breeds) of dogs [28]. It may be valuable to consider closer analysis of the 33 OS-associated loci from dogs, in human targeted studies and search for additional evidence to explain the observation and tolerance of the complex cancer landscapes in OS cells in both species.

The clinical presentation, biologic behavior, histopathology, response-to-treatment, and genetics of OS in dogs are very similar to people [31,32,33], emphasizing the relevance of dogs to serve as a comparative model. Based upon these unique similarities, dogs that develop OS spontaneously have the capacity to serve as accurate tumor models for deepening our fundamental understandings of OS etiopathogenesis and biology. This review article highlights the commonalities shared between people and dogs with respect to environment, bone metabolic and genetic factors which participates in OS etiopathogenesis, and exemplifies how the study of comparative oncology can advance bone sarcoma research.

## 2. Comparative Environmental Exposures and Osteosarcoma

### 2.1. Ionizing Radiation—Human

Ionizing radiation is a well-recognized carcinogenic agent and the epidemiologic association between cancer development and therapeutic radiation exposure have been thoroughly described [34,35,36]. However, the complex and inter-related molecular mechanisms responsible for radiation-induced cancer formation remain incompletely characterized, but minimally involve radiation-induced DNA damage with the subsequent generation of irreparable somatic mutations in non-cancerous, by-stander cells. Specifically for radiation-induced bone sarcomas, such as OS, induction of DNA damage to resident mesenchymal stem cells or pre-osteoblasts by ionizing radiation results in cellular “initiation”, the first and necessary step towards the process of malignant transformation. 

The mutagenic capacity of ionizing radiation is a definitive etiologic factor associated with OS development [37,38,39,40,41,42]. Interestingly, some of the earliest epidemiologic evidence linking radiation exposure and OS formation was first noted in American radium dial painters, who ingested large quantities of radium by licking the tips of paintbrushes containing radium paint used for manufacturing fluorescent watches. As a consequence to excessive and prolonged radium exposure within the oral cavity, American dial painters developed pathologic bone conditions with high frequency including jaw necrosis, osteitis, and OS [43,44]. Likewise in Germany during the 1950s, the use of intravenous radium-224 chloride, a short-lived alpha-particle emitter, was prescribed to alleviate the painful symptoms associated ankylosing spondylitis [45]. Although effective for treating ankylosing spondylitis, subsequent epidemiologic studies implicated radium-224 chloride exposure with the development of OS [46,47,48]. In addition to radium, historically, other medicinal radioisotopes have also been associated with OS formation, including Thorotrast, a liquid suspension containing thorium dioxide used as a radiocontrast agent in the early 1930–40s. The induction of OS secondary to Thorotrast exposure was secondary to prolonged exposures of trabecular skeletal sites to ionizing alpha-radiation emissions [49,50]. Collectively, epidemiologic and observational studies derived from the use of radium and other radioisotopes in people firmly establish the link between ionizing radiation exposure and bone sarcoma pathogenesis. 

Despite initial risk factors associated with radioisotope exposure and OS development, more recent investigations have focused on the relationship between the genesis of secondary malignant neoplasms, such as OS, following definitive treatment of primary childhood cancers with therapeutic radiation therapy alone or in combination with cytotoxic agents [37,38,41]. In order to be categorized as a secondary malignant neoplasm, post-radiation OS must satisfy the modified Cahan criteria being: (1) the absence of histologic or radiologic evidence of bone pathology (non-malignant or divergent malignancy) prior to therapy; (2) the development of OS in an irradiated area; and (3) a minimal latency period three to four years from radiation therapy to development of OS [51,52]. Based on three large cohort and case-control studies conducted between the years of 1980–2000 [37,38,41], investigative findings consistently support the association between therapeutic radiation exposure and secondary OS development. The cumulative incidence of developing OS over 20 years is reported to be 0.9%–2.8% following definitive treatment for all types of pediatric tumors. However, treatment of certain primary tumor histologies resulted in significantly higher incidences of secondary OS, and included heritable retinoblastoma (7.2%–12.1%), Ewing’s sarcoma (5.4%–6.7%), and other malignant bone or soft tissue sarcomas (2.4%–2.5%) [37,38]. Similarly, the relative risk of OS was reported to be 43–133 times higher in the affected cohort compared with that of the general population [37,38,41]. Finally, the majority of studies support an increased risk for OS development with increasing cumulative dose of radiation delivered to the affected bone, as well as, dosage of alkylating agent systemically administered [37,38,41].

Although secondary OS has been associated with high doses of ionizing radiation from therapeutic or occupational-related exposures, the development of OS following chronic exposure to lower doses of environmental radiation remains poorly characterized and speculative. Recent epidemiologic evidence derived from long term follow up of atomic-bomb survivors of Hiroshima and Nagasaki suggest that radiation-induced bone sarcoma development, including OS, may be associated with much lower doses of ionizing radiation than previously reported [53]. A total of nineteen cases of bone sarcoma (OS, n = 5) were identified among 80,181 Japanese subjects meeting study inclusion criteria. The incidence of bone sarcoma development was 0.9 per 100,000 person-years, and average time from radiation exposure to bone sarcoma diagnosis was 29.3 ± 12.1 years. Interestingly, a dose threshold was identified at 0.85 Gy with a linear dose-response association above this threshold (relative risk of 7.5 per Gy in excess of 0.85 Gy). The findings from this study suggest that acute exposures to even very low levels of ionizing radiation may increase the risk for secondary bone sarcoma development [53].

### 2.2. Systemic Ionizing Radiation—Dog 

Dogs have been used as large mammalian models to assess the potential risks associated with OS development following exposure to radioactive isotopes, particularly plutonium and strontium-90. Plutonium is a byproduct of nuclear fission, with various plutonium isotopes differing in half-life and energy level. Plutonium-239 is the isotope most useful for the production of nuclear weapons, while plutonium-238 is another isotope that emits alpha particles and is used as a heat source in radioisotope thermoelectric generators. Strontium-90 is also a byproduct of nuclear fission, and can be found in spent nuclear fuel, radioactive waste, and nuclear fallout from nuclear tests. Strontium-90 possesses osteotropism, and after entering a living organism, approximately 20%–30% of strontium-90 is deposited within the bone and bone marrow.

The effect of plutonium exposure in dogs and subsequent OS development has been studied to explain a high rate of cancer-associated mortalities in Mayak metallurgical and radiochemical plutonium plant workers [54]. The Mayak plant is a large nuclear facility in Russia, and historically served as part of the Soviet Union’s nuclear weapons program. In 1957, the Mayak plant experienced a catastrophic nuclear accident with the release of high-level radioactive waste into the surrounding territories. Following the deposition of radioactive wastes into the environment, human observational studies demonstrated that Mayak nuclear plant workers experienced higher rates of solid tumor development, particularly involving lung, liver and bone in which plutonium concentrates [55,56,57].

To better understand the carcinogenic effects of plutonium exposure in people, lifespan investigations were conducted in beagle dogs following single exposure to plutonium isotopes either intravenously or by aerosolization; and these fundamental investigation generated valuable information regarding the incidence and distribution of plutonium-induced OS [58,59,60,61]. In one report, intravenous plutonium-239 citrate was administered to dogs and achieved an average skeletal dose of 1.3 Gy, which resulted in the development of OS in 76 out of 234 beagles [58]. In another study where beagle dogs were exposed to plutonium dioxide via aerosolization, skeletal tissue received alpha-particle doses ranging from 0.08–8.7 Gy, which resulted in OS development in the majority of beagle dogs, specifically 93 out of 144 (65%) [61]. 

In addition to describing the incidence of plutonium-induced OS in beagles, other studies have characterized the skeletal distribution of OS associated with plutonium exposure. Unlike naturally-occurring OS in people and dogs, whereby the majority of OS lesions arise from the metaphyseal regions of appendicular weight-bearing bones, plutonium-induced OS preferentially affected the axial skeleton within regions of high bone turnover and vascularity [59,60]; accounting for 50%–69% of all OS lesions identified radiographically. Based upon the observed skeletal site for OS formation (axial) following plutonium exposure in dogs, and the similar skeletal distribution pattern of OS reported in Mayak nuclear plant workers, observational evidence derived from beagles supported the hypothesis that OS development in Mayak workers were a consequence of occupational plutonium exposure.

Similar to studies with plutonium, the bone tumorigenic effects of strontium-90 in beagle dogs has also been investigated through lifespan observational studies to assess the risk of OS development in people inadvertently exposed to environmental radioisotope contamination associated with nuclear power plant accidents [62,63,64]. In beagle dogs subjected to strontium-90 aerosolization, 45 primary bone tumors developed in 31 out of 66 dogs exposed. The majority of primary bone tumors were OS (60%), however other tumor histologies included hemangiosarcoma (31%), chondrosarcoma (7%), and myxosarcoma (2%) [65]. In a separate study, a dose-dependent tumorigenic effect was observed in beagle dogs fed strontium-90, with 66 primary bone tumors developing in 43 out of 403 dogs exposed. Again, the most frequent tumor histology was OS, accounting for 74% of all bone tumors identified. Unlike plutonium, which preferentially induces OS formation within the axial skeleton (69%) [60], beagle dogs fed strontium-90 developed OS predominantly in appendicular sites (74%) [66], more similar to the skeletal distribution of naturally-occurring canine OS.

### 2.3. Localized Ionizing Radiation—Dog

To establish dose tolerance guidelines and assess the risks for secondary malignancy development following therapeutic radiation in people, the National Cancer Institute conducted a series of experiments using dogs as radiobiologic models to assess acute and late toxicity, as well as the carcinogenic effects of intraoperative radiation therapy. Over a 15-year trial, 238 dogs were treated with 12 different intraoperative radiation therapy protocols, with 59 dogs having long-term follow up of greater than two years post radiation. Nine dogs developed secondary malignancies within the intraoperative radiation therapy field, and OS comprised 33% of these secondary tumors. The median latency period for secondary malignancy formation was 40 months and the median intraoperative radiation dose associated with tumor development was 30 Gy [67,68]. In accordance with the National Cancer Institute studies, a separate investigation conducted in normal beagle dogs also demonstrated the induction of OS following either intraoperative radiation therapy alone, or in combination with external beam radiation therapy [69]. Based upon study findings, a dose relationship and additive carcinogenic effect for OS induction following a single intraoperative radiation dose combined with fractionated external beam radiation was demonstrated. Four to five years post irradiation, seven out of 27 dogs (25%) treated with combination intraoperative radiation therapy and external beam radiation therapy developed OS within the treatment field [69]. 

Similar to people who develop secondary OS following definitive treatment of primary childhood cancers with therapeutic radiation therapy, cancer-bearing pet dogs treated with therapeutic megavoltage radiation for long-term control of non-osseous neoplasms also develop OS secondarily [69,70,71]. The percentage of cancer-bearing pet dogs treated with external beam radiation therapy that eventually develop secondary OS within the radiated field remains low (3.4%–8.4%) with a reported wide latency period of 1.7 to 8.7 years post radiation [69,70,71]. 

## 3. Comparative Skeletal Growth and Osteosarcoma

### 3.1. Skeletal Growth—Human

Physiologic and pathologic perturbations in homeostatic bone turnover appear to participate in the etiopathogenesis of OS. Several lines of clinical evidence support the role of accelerated bone remodeling as a contributing factor for OS formation. First, OS has a bimodal age distribution, with the largest incidence of OS occurring in adolescents between 10–19 years of age, which correlates with the time of peak pubertal skeletal growth [9,10,72]. Although the overall incidence of OS is slightly greater for males than females across all age groups, it has been reported that females less than 15 years of age have slightly higher rates than males in the same age group, likely due to the earlier onset of puberty and consequent skeletal maturation in females [73]. Second, OS in adolescents predominantly develops in the lower long bones, specifically within the metaphyseal regions of trabecular bone adjacent to growth plates. The most common anatomic sites of involvement include the distal femur and proximal tibia which comprise the major weight-bearing stifle joint, and less frequently the proximal humerus [7,10]. Based upon the premise of bone functional adaptation in response to mechanical loading [74], major weight-bearing bones of the lower extremities will experience greater rates of bone remodeling, which reinforces the relationship between skeletal growth and OS formation. To further substantiate the link between skeletal growth and the development of OS, several epidemiologic studies suggest that height is a risk factor for developing OS [75,76,77]. Adolescent patients diagnosed with OS tend to be taller than average, however, this association between height and OS risk only relates to patients diagnosed with OS earlier than 18 years of age [78].

In addition to accelerated homeostatic bone turnover, dysregulated skeletal remodeling also participates in OS etiopathogenesis. Individuals diagnosed with Paget’s disease of bone or other skeletal dysplastic syndromes are at increased risk for developing adult-onset OS [79]. Paget’s disease is a chronic skeletal disorder marked by focal areas of dysregulated and excessive bone resorption and formation, ultimately resulting in the disorganized deposition of lamellar bone, excessive fibrous connective tissue, and consequent marrow hypervascularity [80,81]. The incidence of OS secondary to Paget’s disease is not precisely known, however, it is estimated that approximately 1% of patients with Paget’s disease will go on to develop OS [79,82]. Significantly for elderly patients, pre-existing Paget’s disease accounts for 20% of OS development in patients older than 40 years, and up to 50% of OS formation in patients older than 60 years [83,84]. Finally, other pre-existing bone abnormalities and benign skeletal neoplasms which result in altered bone remodeling such as solitary or multiple osteochondromas, solitary enchondroma or enchondromatosis, multiple hereditary exostoses, fibrous dysplasia, and chronic osteomyelitis are associated with an increased risk of OS development, which further underscores the association between bone remodeling and OS formation [85,86,87,88].

### 3.2. Skeletal Growth—Dog

Similar to people, bone formation and remodeling also appear to participate in the etiopathogenesis of OS in dogs. First, OS classically affects large and giant breed dogs, often diagnosed in the Saint Bernard, Great Dane, Rottweiler, German shepherd, and Golden retriever breeds. Increasing weight and height appear to be important risk factors for OS development, with dogs weighing more than 40 km accounting for 29% of all OS cases, and only 5% of OS occurring in dogs weighing less than 15 km [89]. Second, although more common to affect geriatric dogs, canine OS predominantly develops in the metaphyseal regions of major weight-bearing long bones, with 75% of OS originating from the appendicular skeleton [90,91]. Given the plantigrade locomotion and anatomy of canids, skeletal weight distribution is divided unequally between forelimbs (70%) and hindlimbs (30%). Correlating with skeletal load forces and bone remodeling activities, OS develops in the forelimbs and hindlimbs at a 2:1 ratio, with the most common skeletal sites being the distal radius and proximal humerus [92]. Similar to people, pre-existing skeletal abnormalities and associated dysregulated bone remodeling occurring in dogs also participates in OS etiopathogenesis. Clinical descriptive reports which support this association include the development of OS at prior skeletal sites of metal implants or internal fixatives used for fracture repair, chronic osteomyelitis, and bone infarcts [93,94,95,96,97,98,99,100,101]. 

## 4. Comparative Genetic Pathogenesis and Osteosarcoma

### 4.1. Genetic Pathogenesis—Human

The evidence implicating genetic factors in OS development is supported by familial cancer predisposition syndromes, whereby germline or somatic defects in genes encoding either tumor suppressor proteins or RECQ helicase enzymes have been associated with increased incidences of OS [10,102]. Of the tumor suppressor genes which are involved in pediatric OS development, derangements in *P53* and retinoblastoma (*RB*) have been most thoroughly characterized [102]. 

The tumor suppressor gene, *P53*, is critically involved in DNA repair, cell cycle arrest, and programmed cell death [103]. As such, mutations in *P53* predispose to the development of cancer via global genomic instability and dysregulated cell cycling [104,105]. Li-Fraumeni syndrome is a rare autosomal dominant hereditary disorder linked to germline mutations of the *P53* tumor suppressor gene [106]. People diagnosed with Li-Fraumeni syndrome are predisposed to develop various malignancies at a young age, including tumors of the breast, soft tissue, adrenocortex, brain, hematopoietic system, as well as OS [107,108]. OS is reported to be the second most common cancer developing in patients diagnosed with Li-Fraumeni syndrome, and germline mutations in *P53* are responsible for 3% of all OS diagnosed in children [109]. Although the incidence of OS development secondary to germline *P53* mutations is relatively low, in sporadic OS arising in older patients, the frequency of *P53* mutations range from 40 to 60% in high-grade tumors [110,111,112]; suggesting that *P53* is not only involved in OS formation, but also participates in OS progression. Several different *P53* mutations have been identified in OS including point mutations, gross gene rearrangements, and allelic loss [110,111,113]; however, the most common OS-associated *P53* gene aberrations are missense mutations within exons 5–8 [110], which are responsible for *P53*’s binding capacity to DNA and exertion of regulatory transcriptional activities.

Another major tumor suppressor gene involved in the etiopathogenesis of OS is the retinoblastoma (*RB*) gene. The retinoblastoma protein (RB) belongs to a family of pocket proteins including p107 and p130, which regulates cellular progression through G1 phase of the cell cycle by virtue of their phosphorylation status [114,115]. As such, mutations in the *RB* gene result in dysregulated cell cycling and differentiation, with consequent predispositions for cancer development. Patients with hereditary retinoblastoma (*RB*) have a germline mutation in a parental *RB* allele, which predisposes to development of multifocal and bilateral retinoblastomas at a young age. Long-term survivors of hereditary retinoblastoma have an increased incidence of OS development later in life, equivalent to 500 times the incidence of OS compared to the normal population [116,117,118]. Interestingly, the standardized incidence ratio for OS in patients diagnosed with hereditary retinoblastoma and treated with radiation (406-fold) is significant higher than in patients not receiving radiation therapy (69-fold); observational findings which strongly support the interplay of genes and environment in OS pathogenesis [119]. In addition to hereditary retinoblastoma, somatic loss of *RB* is frequently found in sporadic OS, as evidenced by loss of heterozygosity (LOH) on chromosome 13q14 in 60% of OS tumors [120].

In addition to mutations in tumor suppressor genes, other inherited, cancer-prone disorders have also been identified to increase the risk for OS development. RECQ helicase is a family of helicase enzymes important for genome maintenance, and are necessary in eukaryotes for high fidelity DNA replication [121,122]. RECQ helicase activities suppress spontaneous and damage-induced chromosomal recombination events, and therefore minimize the likelihood for malignant transforming events. Mutations in specific human *RECQ* genes are implicated in heritable diseases including Rhothmund-Thomson, Werner, and Bloom syndromes [123]. These syndromes are associated with a high incidence of chromosomal abnormalities, which predispose to a variety of pathologies, including cancer formation, such as OS [123]. Rhothmund-Thomson syndrome is an autosomal recessive condition characterized by dermatologic and skeletal pathologies, with consequent increased risk for developing OS. Based upon one cohort study which evaluated loss of function mutations in the *RECQL4* gene in patients diagnosed with Rhothmund-Thomson syndrome, 13 out of 41 patients developed OS [124]. Werner and Bloom syndromes are two other *RECQ* helicase disorders, which predispose to the development of various cancers, including OS [122,125,126]. However the incidence of OS associated with Werner and Bloom syndromes is lower than with Rhothmund-Thomson syndrome. Overall, gene mutations in the *RECQ* helicase family is associated with very low incidences of OS, and accounts for a minuscule percentage of OS diagnosed annually.

### 4.2. Genetic Pathogenesis—Dog

Although not as well characterized as in people, ample evidence exists implicating the involvement of genetic and heritable factors for the development of OS in dogs. Currently the most thoroughly described gene mutation, which contributes to OS formation and/or progression in dogs, is *P53* [127,128,129,130,131,132,133,134]. Initial studies performed in immortalized canine OS cell lines demonstrated that the functionality of the *P53* gene was defective based upon the incapacity of *P53* to regulate appropriately the transcriptional expression of downstream target genes including *P21* and *MDM2* following genotoxic insult [129]. Furthermore, *P53* mRNA and protein were overexpressed in 60% of cell lines, and correlated with the presence of missense point mutations within the DNA-binding domain [129]. In corroboration with initial cell line studies, mutations in *P53* have also been demonstrated in dogs with spontaneously-arising OS. Several studies using either single-strand conformational polymorphism, polymerase chain reaction, or Southern blotting, followed by nucleotide sequence analysis have identified missense mutations involving exons 4–8 of *P53* in 24%–47% of all spontaneously-arising OS samples [127,131,133,134]. In addition to exons 4–8, the entire gene sequence of *P53* has also been assessed by polymerase chain reaction and single-strand conformational polymorphism from 59 spontaneously-arising appendicular and axial OS samples [128]. In 24 OS tumors, *P53* mutations were identified with most gene abnormalities located in exons 4 and 5; however, two mutations were located in a non-coding region of the *P53* gene, and one mutation was identified in exon 9. The majority of *P53* gene abnormalities were point mutations (74%) which resulted in an amino acid substitution, will a lesser percentage of mutations (26%) being deletions [128]. Finally, through the implementation of targeted microarray-based comparative genomic hybridization analysis of 38 canine OS cases, similar recurrent cytogenetic aberrations classically present in human OS samples were also identified in OS specimens collected from dogs, including LOH of the *P53* gene in 18% of tumors [135].

Substantiation for the presence of *P53* mutations in sporadic canine OS has also been documented by immunohistochemical studies, as a hallmark of many *P53* mutations is enhanced protein stability of this normally labile protein, enabling detection of protein with methodologies, such as immunohistochemistry [136]. In one study evaluating P53 protein expressions in 106 osteogenic tumors, a greater percentage of appendicular (84%) OS overexpressed P53 protein in comparison with OS arising from the axial skeleton (56%) and other non-OS bone tumors (20%) [132]. Finally, loss of *P53* gene function in 167 osseous tumors has been characterized by P53 nuclear staining frequency and intensity expressed as a P53 index. Of 103 OS samples, 67% stained positively for P53 protein, and the P53 index was significantly greater in OS derived from the appendicular (n = 84) *vs.* axial (n = 38) skeleton [130]. Interestingly, P53 index of appendicular OS derived from Rottweilers was significantly higher than Great Danes or other commonly affected breeds, supporting the notion that *P53* gene mutations may be associated with breed susceptibilities to OS development [137].

Another tumor suppressor gene likely to be permissive for the development of OS in the dog is the *RB* gene. Although germline *RB* gene mutations have not been documented in canines, sporadic somatic *RB* gene mutations are likely responsible for the development of unilateral retinoblastoma infrequently documented in dogs [138,139]. Based upon investigations using five tumorigenic immortalized canine OS cell lines, the *RB* gene signaling pathway was dysregulated with the persistence of hyperphosphorylated RB protein in the absence of mitogen stimulation. Despite apparent aberrant *RB* gene signaling, reduction in RB protein was only identified in one of five cells lines [129]. Corroborating these *in vitro* findings, the evaluation of 21 spontaneously-arising OS failed to identify gross *RB* gene alterations by Southern blotting, and protein expressions of RB were identified in all OS samples evaluated [131].

Despite normal protein expression of RB in canine OS samples, the observed translational normalcy does not exclude the possibility for allelic deletion of the *RB* gene, as prior studies in human OS samples have demonstrated that LOH at the *RB* gene locus does not absolutely correlate with inactivation of the *RB* gene at the protein level [120]. Substantiating the possibility that *RB* gene may be have allelic deletion in spontaneously-arising canine OS, analysis of 38 OS samples with comparative genomic hybridization techniques identified copy number loss in 11/38 cases (29%), resulting in a correlative reduction or absence of *RB* protein expression in 62% of OS samples tested [135]. Based upon these recent investigative findings, it is probable that aberrations in the *RB* gene indeed participate in sporadic OS formation and/or progression in dogs.

Inherited, cancer-prone disorders have not been thoroughly characterized in dogs, therefore it is uncertain if defects in the RECQ helicase genes predisposes to OS formation in canines. Nonetheless, a growing body of evidence in dogs supports breed-associated inheritance of OS, especially in Scottish Deerhounds, Rottweilers, Greyhounds, Great Danes, Saint Bernards, and Irish Wolfhounds [137,140,141,142,143,144]. Given that many domestic dog breeds have narrow genetic diversity as a consequence of selective breeding practices; this has provided the opportunity to more clearly elucidate the heritability of OS in dogs. For Scottish Deerhounds in particular, the reported incidence of OS formation is 15% [140,141], and has been shown that the narrow heritability in this breed was 0.69; indicating that 69% of the cause for OS development in Scottish Deerhounds is due to heritable trait, likely a Mendelian major gene with dominant expression [141]. Further studies in Scottish Deerhounds using of a whole genome linkage approach have mapped a novel locus (OSA1) for OS formation in this breed to CFA34, and provides the opportunity to pinpoint specific candidate genes directly involved in OS etiology for not only dogs, but humans alike. Promisingly, the region of interest on CFA34 is syntenic to human chromosome 3q26, which is associated with a high incidence of LOH in human OS [145,146], and therefore putatively codes for yet unidentified tumor suppressor genes involved in OS pathogenesis.

## 5. Summary

The etiopathogenesis of OS in humans is complex and remains incompletely characterized; however, likely involves interactions among environmental, bone metabolic and genetic factors. Given the heterogeneous and chaotic nature of OS in people, inroads towards a better understanding of OS etiopathogenesis has been slow, and the opportunity to gain insights into OS etiology and biology would be facilitated through the study of comparative model systems that faithfully and accurately recapitulate the development of naturally-occurring OS. Given the strong similarities shared between humans and dogs with regards to OS biology, in conjunction with the narrow genetic diversity of specific canine breeds, pet dogs with naturally-occurring OS can serve as comparative models with excellent potential for accelerating basic science discoveries pertaining to OS etiopathogenesis. With the recognition of the dog as a unique surrogate OS model, it would be expected that significant progress would be made towards identifying key initiating events involved in the etiopathogenesis and progression of OS in people and dogs alike. 
